# Disseminated scabies in a 2‐month‐old infant

**DOI:** 10.1002/ccr3.6334

**Published:** 2022-09-12

**Authors:** Sima Rasti, Rezvan Talaee, Amir Abdoli

**Affiliations:** ^1^ Department of Parasitology and Mycology and Infectious Diseases Research Center, Faculty of Medicine Kashan University of Medical Sciences Kashan Iran; ^2^ Department of Dermatology, Autoimmune Diseases Research Center, Faculty of Medicine Kashan University of Medical Sciences Kashan Iran; ^3^ Zoonoses Research Center Jahrom University of Medical Sciences Jahrom Iran; ^4^ Department of Parasitology and Mycology Jahrom University of Medical Sciences Jahrom Iran

**Keywords:** disseminated, infant, infestation, Iran, *Sarcoptes scabiei*, Scabies

## Abstract

Scabies is a skin disease caused by the mite *Sarcoptes scabiei*. We report disseminated scabies in a 2‐month‐old girl as well as eczematoid lesions in her mother. The diagnosis was made by skin scraping and microscopic examination of the crusts. The patients were successfully treated with permethrin cream (5%).

## Graphical abstract

We report a case of disseminated scabies in a 2‐month‐old infant. Scabies is a skin disease caused by the mite *Sarcoptes scabiei*. The diagnosis was made by skin scraping and microscopic examination of the crusts. The patients were successfully treated with permethrin cream (5%).

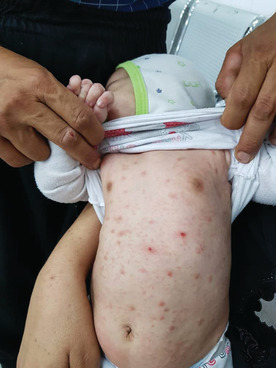

## 1 INTRODUCTION

Scabies is caused by the mite *Sarcoptes scabiei* variety *hominis*. The infestation is transmitted by person‐to‐person contact. *Sarcoptes* mite is burrowed into the stratum corneum of the epidermis. The male mites die after mating and the females begin to lay eggs in the skin burrows.^1^ The common clinical features are erythematous and excoriated itchy papules. Scabies is most prevalent in young children (<18 years).^2^ In infants, the axillae, head and face, diaper region, palms, and soles are more affected.^2^ Scabies could be mimic a broad range of skin diseases.^3^ Hence, differential diagnosis of the infestation should be considered for early treatment and prevention.

## 2 CASE REPORT

**FIGURE 1:**
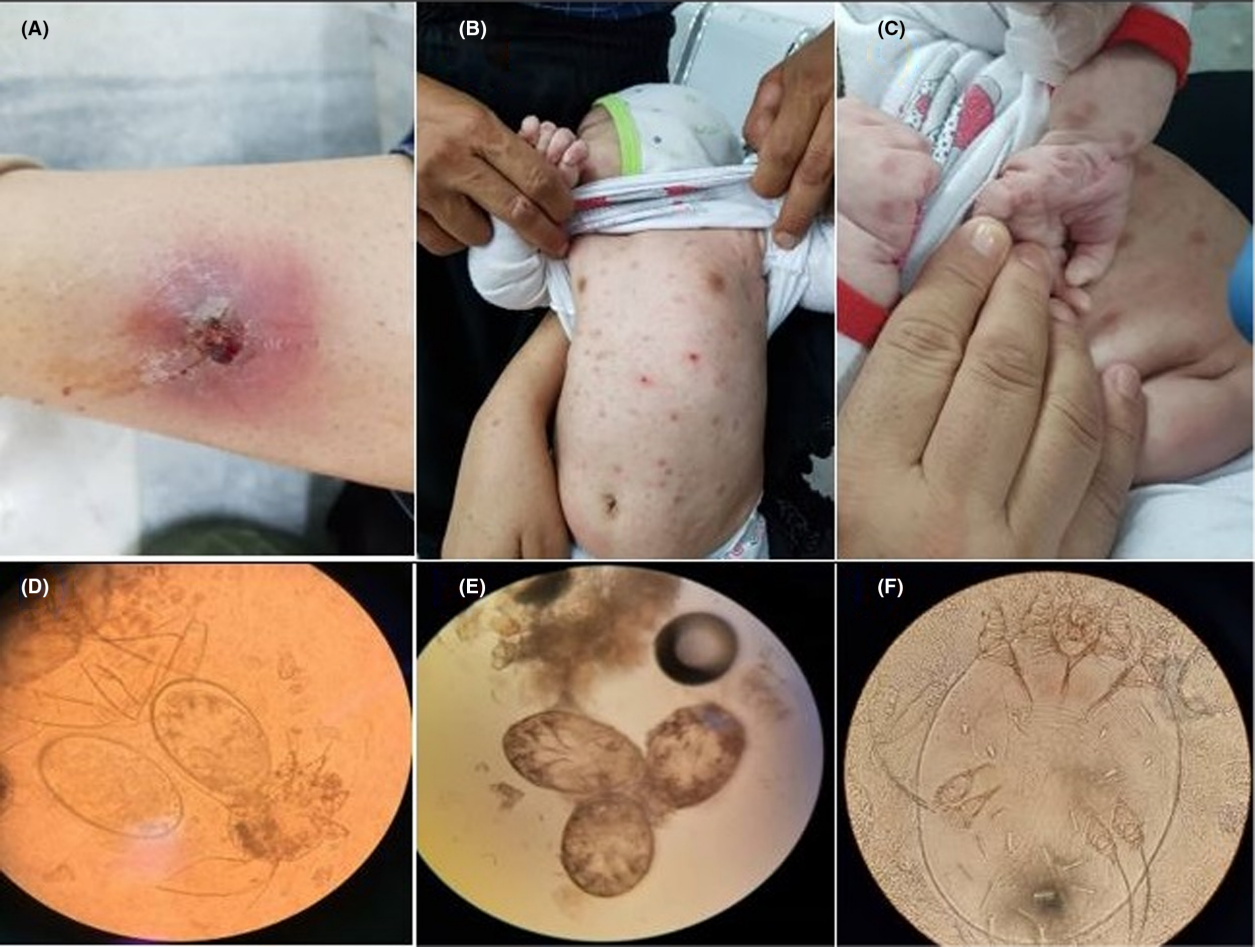
(A) Excoriated papule due to scratching on the forearm of mother; (B) Multiple excoriated papules and erosions on the abdomen of infant; (C) Excoriated papules and erosions on palms and fingers of the infant; (D) Eggs and a nymph; (E) Nymph stage of the mite; (F) An adult female mite

A 38‐year‐old Afghan woman and her 2‐month‐old girl referred to the Shaheed Beheshti Hospital in Kashan (Isfahan Province, center of Iran) presented with a 2‐month history of severe night itching and cutaneous lesions. The mother had eczematoid lesions on her hands. Further examination of her mother revealed eczematous eruptions and excoriations due to scratching on her forearm. The infant had multiple excoriated papules and erythematous lesions on her abdomen, back, legs, and arms, palms and between fingers (Figure 1). Clinical and laboratory assessments of the father revealed no evidence of the infestation. Diagnosis was made by skin scraping. The skin crusts cleared by 10% potassium hydroxide (KOH) solution and visualized under the light microscope. Under the microscope, different stages of the mite (egg, nymph, and adult) were detected. Both the mother and her girl were successfully treated with permethrin cream (5%).

## 3 DISCUSSION

The common clinical features of scabies in infants and young children are vesicles, pustules, and nodules. Eczematization and impetigo are also common. However, lesions may be atypical may confused with acropustulosis or atopic dermatitis. In infants with poor nourishment, pruritus may be so severe and infants can be irritable.^1^, ^2^, ^3^ Scabies should be differentially diagnosed from a variety of pruriginous skin diseases, such as papular urticaria, contact dermatitis, atopic eczema, dermatitis herpetiformis, prurigo nodularis, herpes gestationis, eczema herpeticum, folliculitis/impetigo, and insect bites (e.g., mosquitoes, bed bugs, fleas, and chiggers).^2^, ^4^ However, in infant, differential diagnosis of scabies could be more challenging and the infestation should be differentially diagnosed from infantile seborrhoeic dermatitis (ISD), irritant or contact dermatitis, atopic dermatitis, candidal dermatitis, roseola infantum, pityriasis rosea, viral exanthema, scarlet fever, impetigo, erythema infectiosum, *Tinea corporis* infection, infantile acropustulosis, and nutritional deficiency.^5^, ^6^ Moreover, secondary bacterial infection from *Staphylococcus aureus* and Group A *Streptococcus pyogenes* (GAS) is another complication of the infestation that can lead to acute and superficial bacterial infections, invasive bacteremia, acute post‐streptococcal glomerulonephritis (APSGN) and acute rheumatic fever (ARF).^7^

Skin scraping and microscopic examination is the usual diagnostic method of scabies. Repeated scrapings are needed because the sensitivity is rather low.^1^, ^4^ Topical permethrin 5% lotion is best achieved pharmacological management of pediatric patients with uncomplicated classic scabies.^1^, ^7^ In the complex cases, oral ivermectin is considered safe for treatment.^7^, ^8^ Oral antihistamines can be recommended to alleviate pruritus and post‐scabetic itch.^7^, ^8^

Taken together, differential diagnosis of scabies should be made from other dermatitis, especially in infant and children.

## AUTHOR CONTRIBUTIONS

All authors are involved in the care of the patient. SR examined laboratory assessments. RT performed clinical evaluation and treatment procedures. AA selected the patient images and wrote the manuscript. All authors have approved the final version of the manuscript.

## CONFLICT OF INTEREST

None declared.

## CONSENT

Written informed consent was obtained from the patient's parents for publication of this report and any accompanying images.

## Data Availability

All information of the patient are presented in the manuscript.
